# Laparoscopic repair of Morgagni hernia with extra-abdominal sutures and ileocecal resection for colon cancer: a case report

**DOI:** 10.1093/jscr/rjac572

**Published:** 2022-12-20

**Authors:** Tetsuhiro Hara, Tomohiro Adachi, Kensuke Shimbara, Yuichiro Kai, Naruhiko Honmyo, Ryuta Shintakuya, Noriko Goda, Hideaki Hanaki, Manabu Shimomura, Yoshiro Aoki, Mikihiro Kano, Noriaki Tokumoto, Toshihiko Kohashi, Jun Hihara, Mahito Funakoshi, Shinya Takahashi, Hidenori Mukaida

**Affiliations:** Department of Surgery, Hiroshima City North Medical Center Asa Citizens Hospital, 1-2-1, Kameyama-minami, Asakita-ku, Hiroshima 731-0293, Japan; Department of Surgery, Hiroshima City North Medical Center Asa Citizens Hospital, 1-2-1, Kameyama-minami, Asakita-ku, Hiroshima 731-0293, Japan; Department of Surgery, Hiroshima City North Medical Center Asa Citizens Hospital, 1-2-1, Kameyama-minami, Asakita-ku, Hiroshima 731-0293, Japan; Department of Surgery, Hiroshima City North Medical Center Asa Citizens Hospital, 1-2-1, Kameyama-minami, Asakita-ku, Hiroshima 731-0293, Japan; Department of Surgery, Hiroshima City North Medical Center Asa Citizens Hospital, 1-2-1, Kameyama-minami, Asakita-ku, Hiroshima 731-0293, Japan; Department of Surgery, Hiroshima City North Medical Center Asa Citizens Hospital, 1-2-1, Kameyama-minami, Asakita-ku, Hiroshima 731-0293, Japan; Department of Surgery, Hiroshima City North Medical Center Asa Citizens Hospital, 1-2-1, Kameyama-minami, Asakita-ku, Hiroshima 731-0293, Japan; Department of Surgery, Hiroshima City North Medical Center Asa Citizens Hospital, 1-2-1, Kameyama-minami, Asakita-ku, Hiroshima 731-0293, Japan; Department of Surgery, Hiroshima City North Medical Center Asa Citizens Hospital, 1-2-1, Kameyama-minami, Asakita-ku, Hiroshima 731-0293, Japan; Department of Surgery, Hiroshima City North Medical Center Asa Citizens Hospital, 1-2-1, Kameyama-minami, Asakita-ku, Hiroshima 731-0293, Japan; Department of Surgery, Hiroshima City North Medical Center Asa Citizens Hospital, 1-2-1, Kameyama-minami, Asakita-ku, Hiroshima 731-0293, Japan; Department of Surgery, Hiroshima City North Medical Center Asa Citizens Hospital, 1-2-1, Kameyama-minami, Asakita-ku, Hiroshima 731-0293, Japan; Department of Surgery, Hiroshima City North Medical Center Asa Citizens Hospital, 1-2-1, Kameyama-minami, Asakita-ku, Hiroshima 731-0293, Japan; Department of Surgery, Hiroshima City North Medical Center Asa Citizens Hospital, 1-2-1, Kameyama-minami, Asakita-ku, Hiroshima 731-0293, Japan; Department of Surgery, Hiroshima City North Medical Center Asa Citizens Hospital, 1-2-1, Kameyama-minami, Asakita-ku, Hiroshima 731-0293, Japan; Department of Surgery, Graduate School of Biomedical & Health Sciences, Hiroshima University, 1-2-3, Kasumi, Minami-ku, Hiroshima 734-8551, Japan; Department of Surgery, Hiroshima City North Medical Center Asa Citizens Hospital, 1-2-1, Kameyama-minami, Asakita-ku, Hiroshima 731-0293, Japan

**Keywords:** Morgagni hernia, colorectal cancer, laparoscopic surgery

## Abstract

Morgagni hernia is a rare form of diaphragmatic hernia. It is located at the anterior edge of the diaphragm and does not have an anterior rim. It is difficult to achieve a secure closure and maintain the tension of closure with laparoscopic surgery. We have performed laparoscopic resection of colorectal cancer and hernia repair simultaneously. An 89-year-old woman underwent laparoscopic hernia repair and ileocecal resection simultaneously. Regarding hernia repair, we considered that it would be difficult to use a mesh from the viewpoint of infection due to the colectomy. Therefore, we have done the extra-abdominal suture method. After laparoscopic ileocecal resection, a small incision was made in the epigastric region, and Morgagni hernia repair was performed with extra-abdominal sutures. She had no recurrence of either colon cancer or hernia for 22 months post-operatively. The extra-abdominal suture method can provide secure closure of the hernia orifice for Morgagni hernia.

## INTRODUCTION

A Morgagni hernia is a rare clinical entity and accounts for 3% of all diaphragmatic hernias [1]. Due to the risk of visceral strangulation, surgical repair is recommended. This time, we performed laparoscopic repair of the Morgagni hernia with extra-abdominal sutures and ileocecal resection. The extra-abdominal suture method seemed to be a simple and effective method.

## CASE REPORT

**Figure 1:**
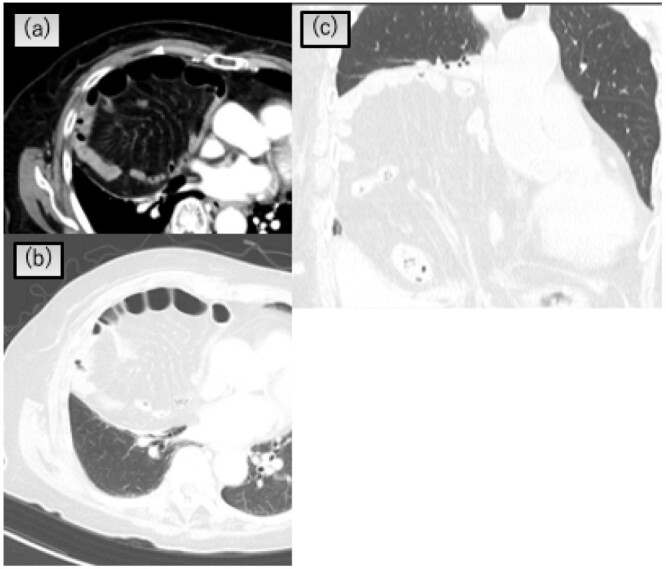
Chest CT scan showing that the transverse colon and greater omentum in the right pleural cavity and atelectasis of the right lower lobe. (**a**) and (**b**) Axial view, (**c**) coronal view.

An 89-year-old woman presented to our department for a positive stool guaiac test. Laboratory investigation, including tumor markers, yielded unremarkable results. Colonoscopy revealed a mass in the ascending colon. Computed tomography (CT) revealed a thickening of the ascending colon and a Morgagni-type hernia with the transverse colon and small intestine trapped in the chest, causing atelectasis of the right lower lobe (Fig. 1a, b, c). The size of the hernia orifice was 48 × 40 mm.

**Figure 2:**
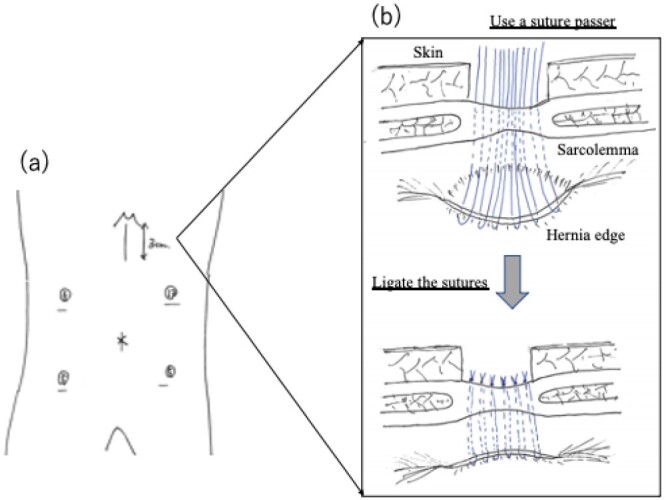
(**a**) Laparoscopic port site position. (**b**) Schematic diagram of surgery. A diaphragmatic defect was closed securely by the extra-abdominal suture method. The defect was closed by placing non-absorbable sutures passing through the full thickness of the anterior abdominal wall and the posterior rim of the defect.

**Figure 3:**
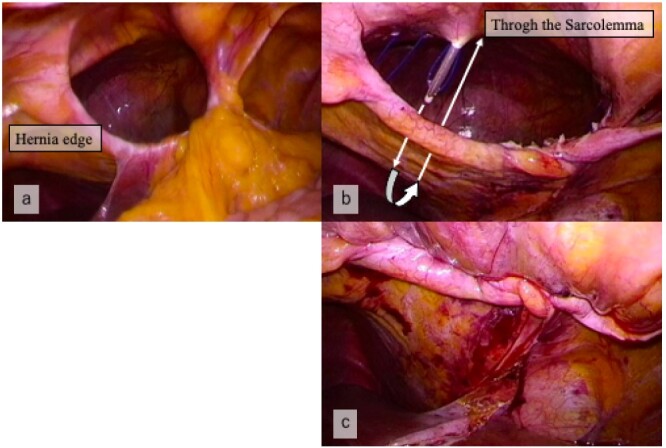
(**a**) Laparoscopic view showing the hernia orifice before suturing. (**b**) The defect closed by the extra-abdominal suture method, using suture passer. (**c**) Laparoscopic view showing closure of the hernia orifice.

The clinical diagnosis was ascending colon cancer complicated with a Morgagni hernia. Based on these findings, we opted for surgical resection. She consented to laparoscopic repair of the diaphragmatic hernia and resection of the ascending colon. Surgery was performed in the supine position with the five ports for ileocecal resection. A large right-sided diaphragmatic defect was found anterior to the liver with the transverse colon and small intestine trapped inside. The hernia contents were gently reduced from the mediastinum. First, laparoscopy-assisted D3 ileocecal resection was performed. We employed an approach using retroperitoneal peeling (i.e. retroperitoneal approach). A 4-cm periumbilical incision including the 10–12-mm port site was made, and the colon was resected, with the creation of an extracorporeal ileocolic anastomosis. After that, we repaired the hernia orifice by extra-abdominal sutures without additional ports. A small incision of approximately 3 cm was made in the epigastric region to expose the sarcolemma (Fig. 2a). After the separation of the hepatic round ligament and falciform ligament of the liver, the anterior and posterior edges of the hernia orifice were pierced using a suture passer. The hernia sac was not resected. The hernia orifice was directly closed with six horizontal mattress sutures using a suture passer. We ligated the sutures outside the abdominal wall to close the hernia orifice (Figs 2b and 3). The surgical time was 292 min, and the total blood loss was 40 ml.

**Table 1:** Reports of laparoscopic repair of adult Morgagni hernia with extra-abdominal suture method in Japan

Case	Author	Year	Age	Sex	Escaped organs	Size (cm)	Recurrence
1	Nakayama	2012	85	Female	Transverse colon, greater omentum	6 × 3	None. (27 months)
2	Hashida	2017	78	Female	Right hemi-colon	5 × 3	None. (37 months)
3	Our case	2022	89	Female	Transeverse colon, small intestine	5 × 4	None. (22 months)

The patient recovered uneventfully. Drainage was required for wound infection, but the patient was discharged 9 days after surgery. She had no recurrence of either colon cancer or hernia for 22 months post-operatively.

## DISCUSSION

This is the first case in which both Morgagni hernia repair without using mesh and colectomy were performed laparoscopically. This is only the eighth case of Morgagni hernia repair with colorectal cancer resection, including laparotomy.

Morgagni hernia was first described in 1769 by Giovanni-Battista Morgagni. It occurs in the sternal triangle on the posterior surface of the sternum. It is rare and accounts for 1–3% of diaphragmatic hernias [1]. It often progresses asymptomatically and is often accidentally discovered during medical examinations. In this case, it was accidentally discovered during the preoperative examination for colon cancer. Due to the threat of serious complications caused by Morgagni hernia, most surgeons agree that surgery is indicated even in asymptomatic patients. There are various approaches to Morgagni hernia repair. A thoracotomy or laparotomy approach is common, but thoracoscopic and laparoscopic surgical procedures are also applied. Compared with traditional open surgical techniques, laparoscopic operation has become the main method to repair Morgagni hernia due to its advantage of minimal invasiveness and fewer complications. In recent years, laparoscopic surgery has often been the first choice.

There are two common methods of repairing the hernia orifice. One is a direct suture closure method, and the other is a mesh method. In the case of open surgery, direct suture closure is often selected, but it requires a high degree of skill to laparoscopically sew and close a hernia orifice that maintains tension. In particular, Morgagni hernia is located on the anterior edge of the diaphragm and does not have an anterior rim, so it is difficult to achieve a secure closure and maintain the tension of closure after suturing the hernia orifice. In recent years, there have been an increasing number of reports of laparoscopic repair using a mesh. Fueyo and Onafowokan performed laparoscopic hernia repair using a mesh in conjunction with colorectal cancer surgery [2, 3], but careful judgment regarding surgical indication from the viewpoint of infection is important. Laparoscopic direct suturing is difficult and often avoided, but the extra-abdominal suture method can be a method to overcome this. The extra-abdominal suture method for Morgagni hernia was first reported by Kuster in 1992 [4]. This technique can maintain tight closure by using rectus abdominis fascia. It can also provide secure closure of the hernia orifice for Morgagni hernia in which the anterior rim is absent. According to reports from Japan, there were two adult cases of diaphragmatic hernia that were treated with the extra-abdominal suture method [5, 6]. Table 1 summarises the two previously reported cases and our case. A total of 27, 37 and 22 months have passed, respectively, and no recurrence has been found in any of the cases.

Generally, the Morgania hernia will be repaired in two stages using a mesh. This case was not selected using the mesh. There were three reasons. One is that the patient was elderly. Second, when the anastomosis is invaginated into the hernia sac, excessive tension to the anastomosis might be happened to the anastomotic leakage. Third, there was a risk of infection due to the colorectal cancer surgery.

In these three reasons, the direct suture closure was preferable, with the extra-abdominal suture method selected. The additional operative time associated with hernia repair was only 30 min, and there was little bleeding. It is considered that the additional invasion was mild. She was discharged according to the clinical path of colorectal cancer and had no recurrence of either colon cancer or Morgagni hernia. We think that the extra-abdominal suture method could be an effective option for laparoscopic hernia repair.

## Data Availability

The dataset generated during the current study are not publicly available but are available from the corresponding author on reasonable request.
